# Synthesis and Study on Ionic Conductive (Bi_1−x_,V_x_)O_1.5−δ_ Materials with a Dual-Phase Microstructure

**DOI:** 10.3390/ma9110863

**Published:** 2016-10-25

**Authors:** Yu-Wei Lai, Wen-Cheng J. Wei

**Affiliations:** Department of Materials Science and Engineering, National Taiwan University, Taipei 106, Taiwan; r00527011@ntu.edu.tw

**Keywords:** solid oxide fuel cell (SOFC), conductor, bismuth oxide, vanadium oxide, electrical conductivity

## Abstract

Homogeneous Bi_2_O_3_-V_2_O_5_ powder mixtures with different amounts of V_2_O_5_ content (≤15 mol%) were prepared by colloidal dispersion and sintering to high density. The sintered and annealed samples were studied by thermal analysis, quantitative X-ray diffraction and scanning electron microscopy. The electrical and ionic conductivities of the conductors were also measured by a four-probe direct current (DC) method. The results of the samples prepared at 600–800 °C and annealed for as long as 100 h show that the sintered samples consisting of a pure γ phase or δ + γ binary phase perform differently in conductivity. The highly conductive δ phase in the composition of Bi_0.92_V_0.08_O_1.5−δ_ enhances the electric conductivity 10-times better than that of the pure γ-sample (Bi_0.94_V_0.06_O_1.5−δ_) between 400 and 600 °C. The compatible regions of the γ phase with the α- or δ phase are also reported and discussed, so a part of the previously published Bi_2_O_3_-V_2_O_5_ phase diagram below 800 °C is revised.

## 1. Introduction

Study of the intermediate-temperature solid oxide fuel cells (IT-SOFCs) has recently gained the attention of material researchers because the working temperature of IT-SOFCs (500–650 °C) is lower, resulting in a longer lifetime of every component and a great reduction in the total cost of the fuel cell (FC) system. Moreover, wider choices of materials (gap-sealing, anode, cathode and housing materials) are being used [1].

The electrolyte, cathode and anode constitute the three-layer structure of fuel cells. Among the layers, the electrolyte has the highest electrical resistivity, forming the highest cell polarization among the components. As a result, searching for appropriate electrolytes with a low ionic resistance is one of the key issues in developing IT-SOFCs.

Bi_2_O_3_ is a well-known electrolyte because of its high electrical conductivity, 1.0 S/cm, in δ phase Bi_2_O_3_ over 730 °C. However, the electrical conductivity drops dramatically below 730 °C to about 10^−3^ S/cm because of the phase transition from the δ phase to the α, β or γ phase [2]. These low temperature phases exhibit poor conductivity and are not appropriate for use as the electrolyte of an IT-SOFC.

To maintain high electrical conductivity below 730 °C, element doping to stabilize the δ phase Bi_2_O_3_ has been tried. The electrical conductivity reduces with increasing dopant concentration, and a secondary phase may appear if the doping content is over the solid-solution limit in Bi_2_O_3_. As a result, finding a minimum doping amount that keeps the phase stable in the single phase is essential for retaining good conductivity. Previous works [3,4,5,6] reported two-element doping, or so-called double stabilizers, reducing the total doping level in Bi_2_O_3_, but enhancing its electrical conductivity. With an appropriate ratio of any double stabilizers, the total doping level could be further reduced and still retain a higher conductivity [5,6].

In the past literature on Bi_2_O_3_-based electrolytes, V_2_O_5_ was shown to be a promising dopant because of its characteristics of multi-valence charge and small ionic radius [7]. One well-known electrolyte in the Bi-V-O system, Bi_2_VO_5.5_, shows an Aurivillius structure, composed of alternative layers of a perovskite layer and rock-salt related layer, and a high electrical conductivity of 0.18 S/cm at 650 °C [8]. In addition, double stabilizers based on Bi_2_VO_5.5_ (e.g., bismuth metal vanadium oxide (BIMEVOX)), with additional oxides of Zr, Mg, Co, Zn and Nb, have been the subjects of many research papers [8,9,10,11,12].

However, there are several discrepancies reported in the literature, i.e., stable phase regions in the published Bi_2_O_3_-V_2_O_5_ phase diagrams, especially in the regions of ≤15 mol% V_2_O_5_ [13,14]. A pure δ phase of the doped Bi_2_O_3_ was the primary concern of our previous work [6,15,16,17,18]. A new dual-phase ionic conductor is proposed in this study. The conductive δ phase is kept as the major composition and composited with other available phases in the B-V-O system. Therefore, this study carefully verifies the solid-solution (single-phase) and two-phase regions, as well as the ionic-electronic conductivity of the Bi-V-O samples.

## 2. Results and Discussion

### 2.1. Dispersion of Bi_2_O_3_ and V_2_O_5_ Powders

**Figure 1:**
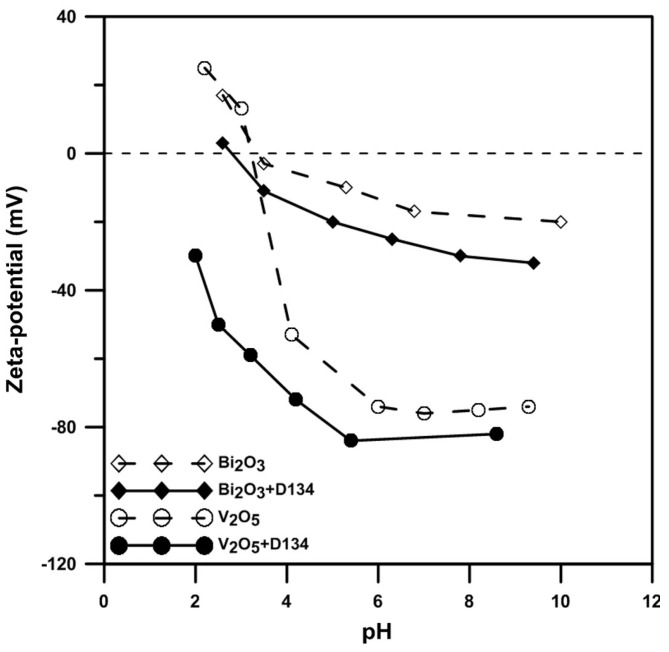
Zeta-potentials of Bi_2_O_3_ and V_2_O_5_ powders with/without 1 wt% D-134 in deionized (DI) water at different pH values.

The zeta-potentials of Bi_2_O_3_ and V_2_O_5_ powders in aqueous solution are shown in Figure 1. A high zeta-potential of the powder ensures a good dispersion condition of the particle in a colloidal state [15]. The isoelectric point (IEP) of V_2_O_5_ in an aqueous solution is located at pH 3.2. When the solution includes additional dispersant D-134 (an ammonium salt of copolymer), the IEP becomes more acidic, at about pH 2.0. The change in the surface potential after the addition of D-134 is possibly due to the following chemical interactions: the V_2_O_5_ powder has a significant solubility 0.8 g/100 mL at 20 °C and pH = 7. Dissolved VO_2_^+^ ions in acidic conditions display a light-yellow color in the slurry. Meanwhile, Coulomb attraction force drives VO_2_^+^ ions to attach on the negative site of the dissociated D-134 polymeric chains, especially under strong acidic conditions (pH < 3.2). As a result, the polymer chain of D-134 in the acidic solution becomes less negative and may strongly adsorb on the charged V_2_O_5_ powder surface and enhance its electric potential to stronger repulsive states.

The zeta-potential of Bi_2_O_3_ powder was investigated by Weng and Wei [15]. The IEP of Bi_2_O_3_ reported in this study is located at pH 3.5, similar to Weng and Wei’s result. The IEP shifts to a more acidic value if the solution contains enough D-134. The surface charging condition of the Bi_2_O_3_ differs from the reported V_2_O_5_ case. We reported that Bi^3+^ dissolved from the Bi_2_O_3_ powder surface is preferentially adsorbed on the negative sites of the D-134 polymeric chain in weak acidic and neutral conditions, altering the ends of the polymer chain to become positively charged. Therefore, the D-134 chain is attracted and anchored on the defective Bi_2_O_3_ surface (which is negatively charged). This is why the surface potential of Bi_2_O_3_ with D-134 changes under neutral/acidic solutions.

### 2.2. Thermal Evolution of Powder Mixtures

#### 2.2.1. Calcination Effect

**Figure 2:**
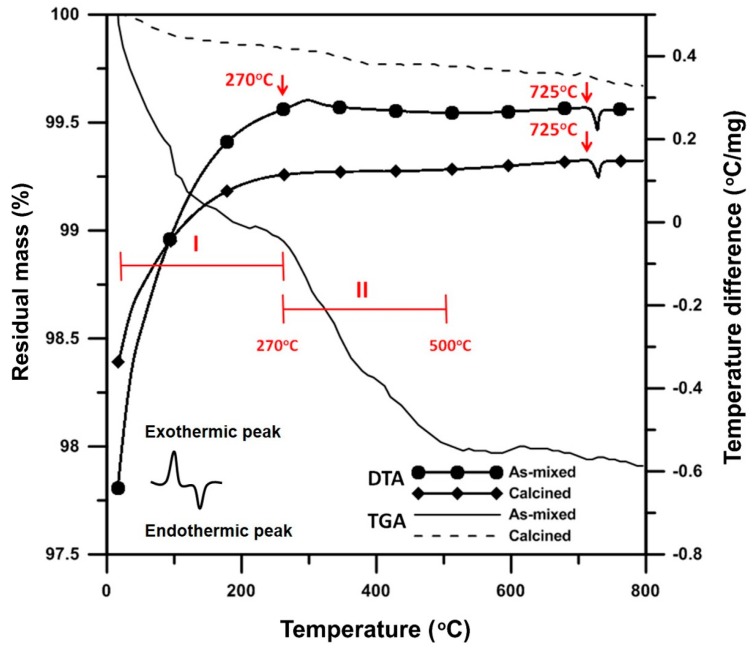
Differential thermal analysis (DTA) and thermogravimetric analysis (TGA) analysis of B1V die-pressed mixture after calcination at 650 °C for 24 h, labeled “Calcined”, or without calcination, labeled “As-mixed”.

**Figure 3:**
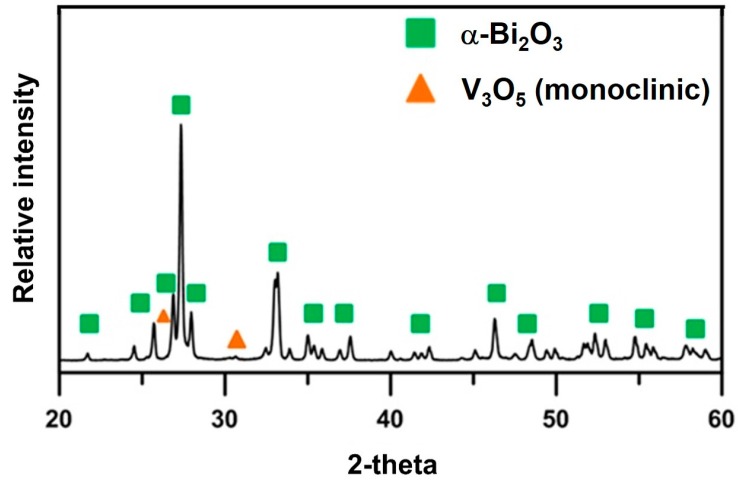
X-ray diffraction (XRD) result of B1V die-pressed mixture after calcination at 350 °C for 24 h. The V_3_O_5_ is indexed by JCPDF 71-39.

In the following sections, the sample Bi_1−x_V_x_O_1.5+x_ is abbreviated as “BxV”, indicating the V_2_O_5_ content is x mol% and Bi_2_O_3_ is (100 − x) mol%. Differential thermal analysis (DTA) and thermogravimetric analysis (TGA) tests were conducted to investigate the thermal behavior of the B1V mixture, which contained only 1 mol% V_2_O_5_. The sample lost 1% of its mass in the Region I (Figure 2) because of the evaporation of both physically- and chemically-absorbed water. At a higher temperature, an exothermic (endo-) peak at 270 °C was detected. This was possibly the burn-out of the polymeric dispersant or possibly the transformation from V_2_O_5_ to V_3_O_5_. Very weak peaks indexed from the X-ray diffraction (XRD) patterns are shown in Figure 3, where the V_3_O_5_ phase possibly appeared in the B1V sample calcined at 350 °C for 24 h, which are the conditions making the exothermic (exo-) peak end. During the reactions, the treatment from 270 to 500 °C resulted in 1 wt% mass loss (Region II in Figure 2). Finally, an endothermic peak at 725 °C was noted, which could be the phase transition from the α- to δ phase inferred from the phase diagram by Kargin and Voevodskii [13]. On the other hand, the samples calcined at 650 °C for 20 h had no exothermic peak at 270 °C and no mass loss below 270 °C, but still showed an endothermic peak at 725 °C. Both results of the as-received and calcined samples showed no endothermic peaks at 690 °C, which was the melting point of V_2_O_5_. As a result, without the calcination step, the V^5+^ ions could have reacted or had diffused entirely into the Bi_2_O_3_ lattice and formed a complete solid solution before melting. As a result, synthesizing Bi_2_O_3_-V_2_O_5_ compounds by directly sintering, skipping the calcination step, is a feasible and energy-saving method.

#### 2.2.2. Phase Evolution

**Figure 4:**
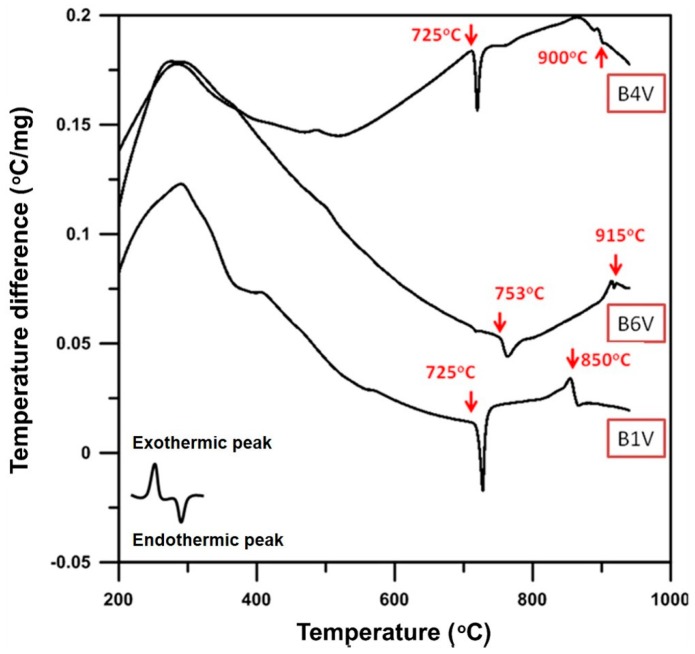
DTA analysis of B1V, B4V and B6V die-pressed mixtures without the thermal process.

The DTA results in Figure 4 reveal that the B1V sample shows two endothermic peaks: one is at 725 °C, which has been pointed out in the previous figure to be the phase transition, the α phase to the δ + γ phases; the other is at 850 °C, which is the melting of the Bi_2_O_3_-rich sample.

Thermal analysis of B4V shows two endothermic peaks; one is at 725 °C, the same as B1V; the other is found at 900 °C, at which the powder melts. B6V has two obvious endothermic peaks, 753 and 915 °C. The temperature 753 °C is the phase transformation points of the γ to δ phase, while 915 °C is the melting point of B6V.

### 2.3. Identification of Crystalline Structures

#### 2.3.1. Solid Solution Range of γ Phase

**Figure 5:**
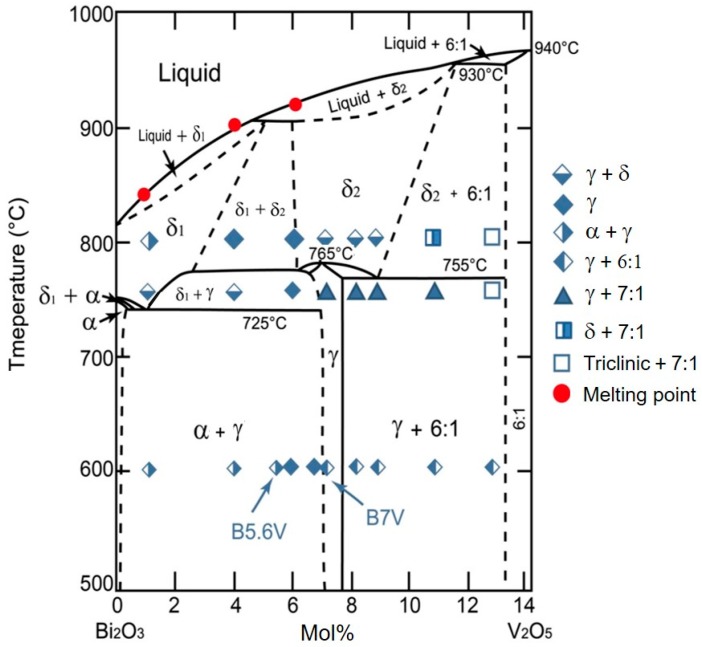
The Bi_2_O_3_-V_2_O_5_ phase diagram with the V_2_O_5_ doping concentration less than 14 mol%. The furnace-cooled phases are plotted in symbol form as originally reported by Kargin and Voevodskii [13].

The γ phase containing 7.0–7.6 mol% V_2_O_5_ had been reported by Kargin and Voevodskii [13], as shown in Figure 5, which also included detailed XRD data and the indexed phases, as shown in Appendix A. The results of the phase information are summarized in additional markers in Figure 5. More than six crystalline phases are identified with V_2_O_5_ content less than 13 mol%.

**Figure A2:**
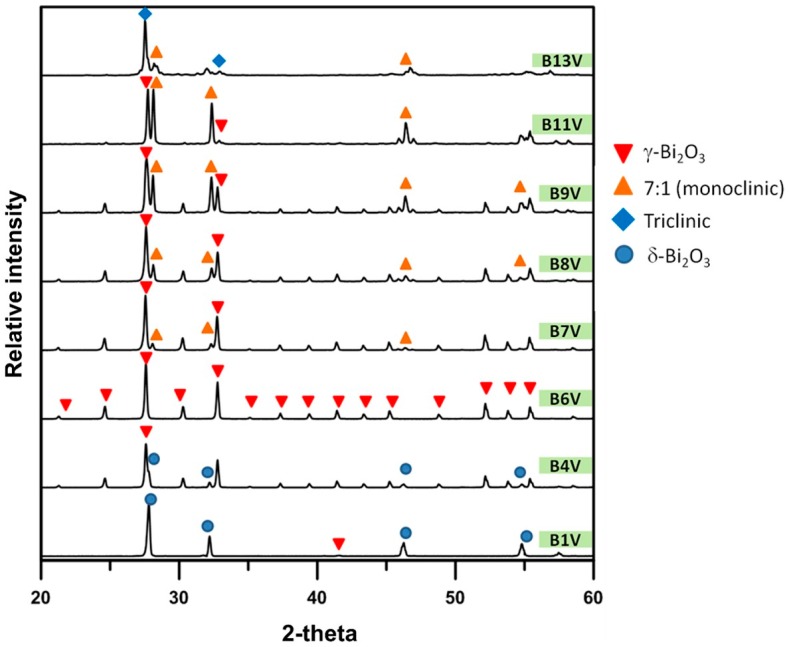
XRD patterns of B1V to B13V die-pressed mixtures, annealed at 740 °C for 100 h.

The phase diagram reported by Kargin and Voevodskii shows α, γ, δ (δ_1_ or δ_2_), 6:1, 7:1 and triclinic phases in the region. To be more precise, the α, 6:1 and 7:1 phases have monoclinic crystal structures; the γ phase has a sillenite crystal structure; the δ phase shows a fluorite crystal structure. The results at 600 °C indicate that the position of the γ phase (5.7–6.9 mol%) is inconsistent with that shown in the diagram of Kargin and Voevodskii. At 600 °C, the phase boundary between the α + γ phases and γ phase is located between 5.6 and 5.8 mol% V_2_O_5_, while the phase boundary between the γ phase and γ + 6:1 phases is located between 6.8 and 7.0 mol% V_2_O_5_. When the temperature increases to 740 °C, an additional phase was found. B4V transformed from the α + γ phases into the γ + δ phases; the samples of B7V–B11V transformed from the γ + 6:1 phases to the γ + 7:1 phases; B13V transformed from the γ + 6:1 phases to the triclinic +7:1 phases; and only B6V retained the same γ phase from 600 to 740 °C. At 740 °C, the phase boundary between the γ + α phases and γ phase is located between 4.0 and 6.0 mol% V_2_O_5_, and the boundary between the γ + 7:1 phases and triclinic +7:1 phases is located between 11.0 and 13.0 mol% V_2_O_5_. Finally, when the temperature increases to 800 °C, the sample of B4V transformed from the γ + δ phases into the γ phase; B7V–B9V transformed from the γ + 7:1 phases into the γ + δ phases; B11V transformed from γ + 7:1 phases into δ + 7:1 phases; and only B6V and B13V remain in the same phases from 740 to 800 °C. At 800 °C, the phase boundary between the γ phase and the γ + δ phases is located between 6 and 7 mol% V_2_O_5_; the phase boundary between the γ + δ phases and δ + 7:1 phases is located between 9 and 11 mol% V_2_O_5_; and the phase boundary between δ + 7:1 phases and triclinic + 7:1 phases is located between 11 and 13 mol% V_2_O_5_. B1V transformed from the α + γ phases into the α + δ phases when the temperature increases from 600 to 740 °C as described before. Note, the B1V sample annealed at 740 °C for 100 h followed by furnace-cooling has a predominant δ phase with a tiny amount of the γ phase, which could be explained if the δ_1_ + γ phases region previously reported by Kargin and Voevodskii is actually wider and the left boundary of the δ_1_ + γ phases is located at a point lower than 1 mol% V_2_O_5_. However, since the relative intensity of the γ phase in the B1V sample annealed at 740 °C, as shown in Figure A2, is too weak to support the second phase appearing in B1V at 740 °C, we only present it here for reference. On the other hand, the B1V sample annealed at 800 °C shows the α + γ phases because the pure δ phase in the 800 °C region is unable to be retained during the furnace cooling.

**Figure 6:**
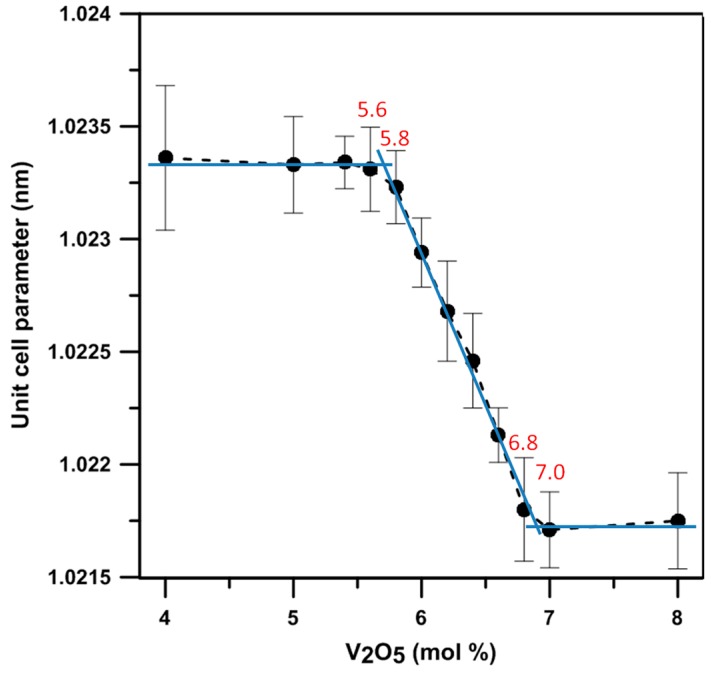
Lattice constants of γ phase (Bi_1−x_,V_x_)_2_O_3_ with the V_2_O_5_ doping levels from 4 to 8 mol%.

The lattice constant of the γ phase in different BxV samples was quantitatively measured in this study using Si as an external standard. The results are shown in Figure 6, which depict the relationship of the lattice constants of the γ phase versus the V_2_O_5_ content. The results indicate the samples with the V_2_O_5_ content between 5.7 and 6.9 mol% show only a single γ phase and a linear change of the lattice constant with the solubility of V in Bi_1−x_V_x_O_1.5+x_, giving two extremes (1.0233 and 1.0215 nm) of the lattice constant of the γ phase. The other research reported by Turkoglu and Belenli [14] claimed the γ phase could be stable between the content of 1 to 7 mol% V_2_O_5_-Bi_2_O_3_ at temperatures <720 °C, and the two-phase co-existing region was between 8 and 10 mol% V_2_O_5_.

The ionic size of V^5+^ (54 pm) is smaller than that of Bi^3+^ (103 pm). The greater the V content in the γ phase, the smaller the unit cell. Out of this range (5.7–6.9 mol%), the α phase (<5.8 mol% V_2_O_5_), 6:1 phase (>6.8 mol% V_2_O_5_) and 7:1 phases co-exist with the γ and form the dual-phase structure, as shown in Figure 5.

#### 2.3.2. Long-Term Mass Loss of BxV Samples

Mass loss of the sintered samples by long-term treatment is our current concern, especially at temperatures close to the melting temperature 825 °C of Bi_2_O_3_ [16]. Four sintered samples were held at 800 °C for 100 h. Only a very small amount of mass loss was detected (Table 1), implying that the annealing of Bi-V-O samples in air did not significantly affect the composition at 800 °C or lower temperatures.

### 2.4. Dual-Phase Conductors

**Figure 7:**
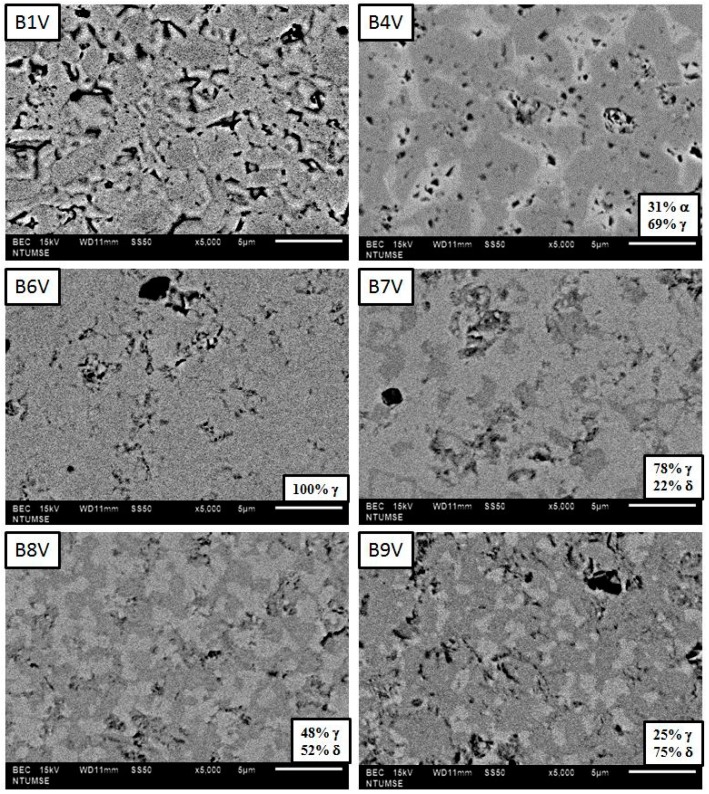
Back-scattered electron (BSE) images of thermal-etched surfaces of B1V–B9V, sintered at 800 °C for 3 h followed by thermal annealing at 680 °C for 100 h. The surfaces have been polished and thermal-etched at 680 °C for 1 h.

Back-scattered electron (BSE) images of B1V–B9V annealed for 100 h are shown in Figure 7 reveal. Four samples, B4V, B7V, B8Y and B9V show a dual-phase structure. The darker contrast region in the BSE images of B7V, B8V and B9V represents a highly conductive δ phase, which contains a higher vanadium concentration as described before in Figure 5, while the brighter contrast region in the BSE images of B6V–B9V represents the γ phase, which contains a lower vanadium concentration, as depicted in Figure 5. Quantitative image analysis was also conducted, and the results are shown in the lower-right corners of the micrographs. The amount of the δ phase (the % in the pictures) increases as the V_2_O_5_ content increased, i.e., the 22 vol% γ phase in the B7V micrograph and 75 vol% δ phase in the B9V micrograph.

B7V shows no continuous δ phase, but B8V does have a continuous δ phase (the bright features) at a quantity of 52 vol%. Similarly, the B9V image shows a large volumetric percentage (75%) in the δ phase. The quantity of the δ phase in B8V and B9V can be retained to room temperature without instantly transforming to the α phase.

Plenty of porosity in B4V is also noted. The α grains of B4V in brighter contrast (Figure 7) are fairly angular and consist of porosity between the γ grains. The porosity in the B4V sample is believed to be a result of the phase transformation of δ grains to α grains below 725 °C, at which the density of the δ phase is clearly lower than the α phase. As a consequence, the electronic and ionic conductivities of dual-phase B8V and single-phase B6V were investigated.

### 2.5. Electric Conductivity

**Figure 8:**
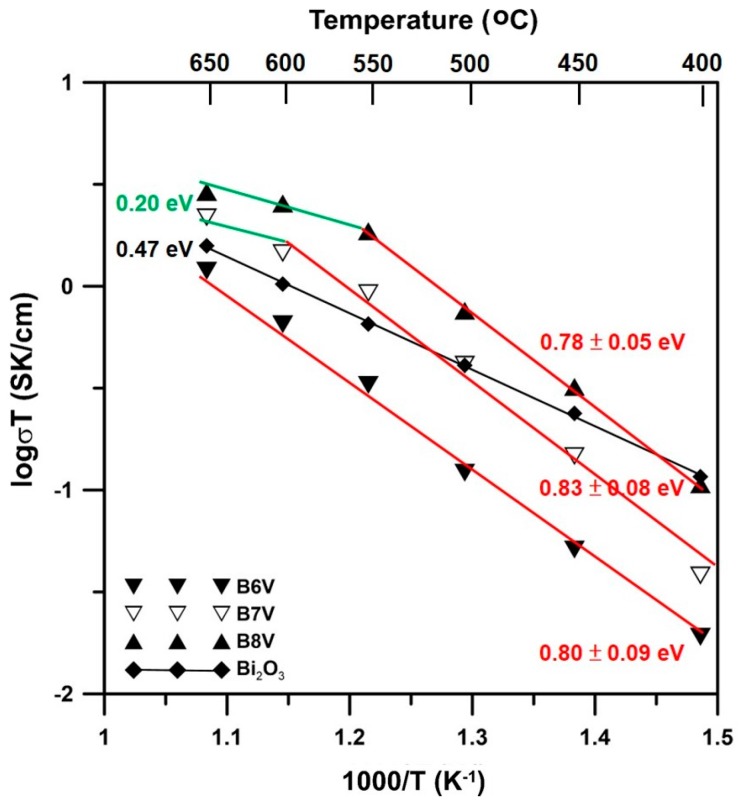
Electrical conductivities and activation energies of B6V, B7V and B8V samples measured by the two-probe direct current (DC) method in air.

The electrical conductivities and activation energies of sintered samples B6V, B7V and B8V are shown in Figure 8. The results measured at 650 °C show that B8V had the highest electrical conductivity, 0.003 S/cm, followed by that of B7V, 0.002 S/cm, and then B6V, 0.001 S/cm.

The conductivity results of B7V and B8V can be fitted to two straight lines with a turning point at 550 and 600 °C, respectively. The slope of each line is fitted by the following equation.

(1) $$\log\sigma T=A-\frac{E_{a}}{RT}$$

where σ is the conductivity (S/cm), *A* is a constant, *E_a_* is the activation energy (eV), *R* is the gas constant and *T* is the testing temperature. The activation energy of B6V of pure γ phase is 0.80 ± 0.09 eV. At temperatures below 550 °C, the *E_a_* values of B7V and B8V are 0.83 ± 0.08 and 0.78 ± 0.05 eV, respectively. The activation energies in the lower temperature region (<500 °C) do not significantly differ. Therefore, the B6V, B7V and B8V samples might have the same conductivity mechanism, oxygen-vacancy-induced conduction, at temperatures below 550 °C. Furthermore, the activation energies of B6V–B7V below 550 °C are higher than that of pure α-Bi_2_O_3_, which is around 0.47 eV, as reported by Takahashi [19]. However, the conductivity mechanism changes to other mechanisms at higher temperatures.

As the temperature exceeds 550 °C, the fitting lines of B7V and B8V obviously bend downward. However, there are insufficient data points in this region to obtain a reliable activation energy value. The value, 0.20 eV, shown in Figure 8, is for reference only.

**Figure 9:**
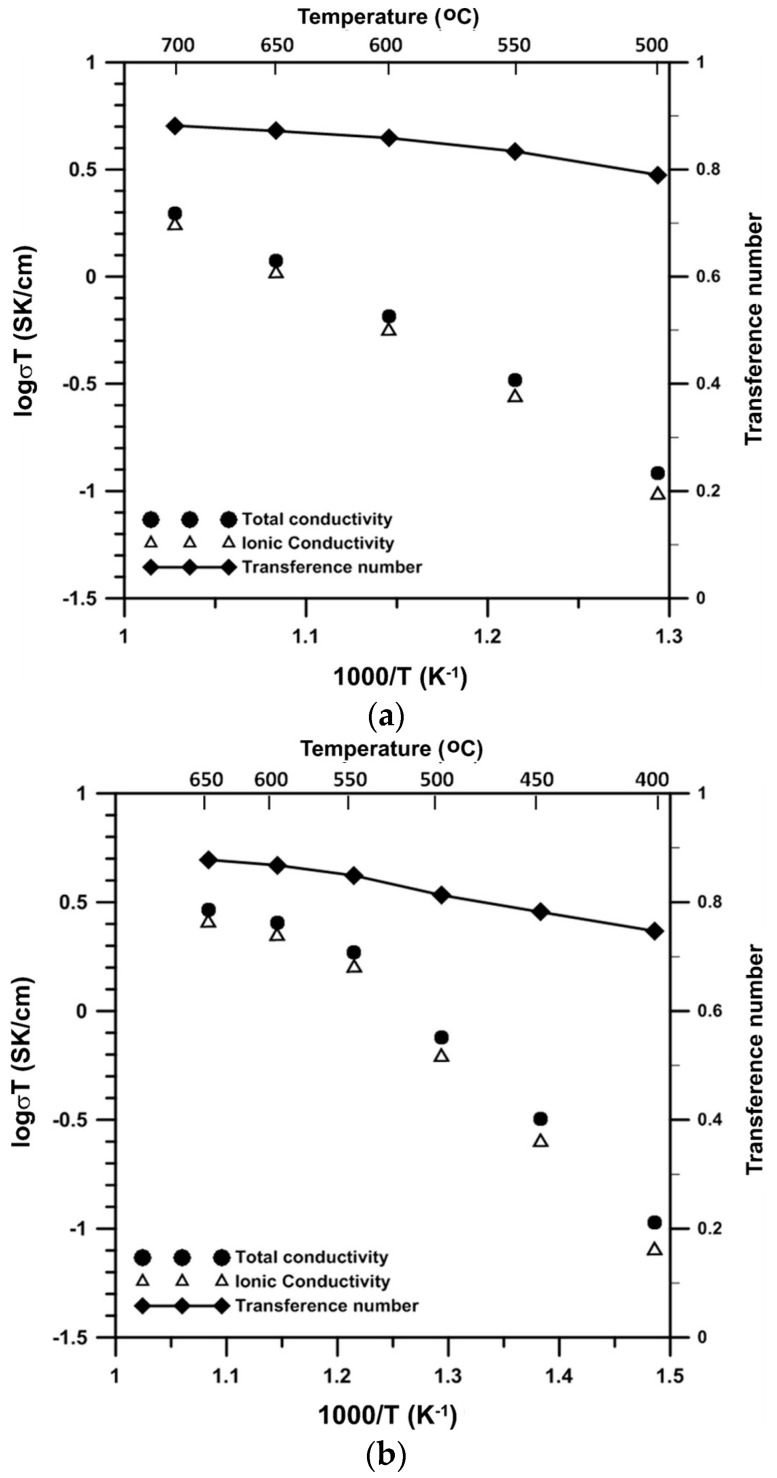
Ionic conductivity, total conductivity and ionic transference number of (**a**) B6V and (**b**) B8V.

The ionic conductivities and transference numbers of B6V and B8V are shown in Figure 9. Both B6V and B8V are mixed ionic-electronic conductors. They have an ionic transference number (*t*_i_) ranging from 0.75 to 0.90. The high transference values of B6V and B8V imply that these materials have a high concentration of ionic carriers and may be suitable as SOFC cathodes. The conductivity of B8V is about three- and 1.7-times higher than that of B6V at 550 and 650 °C, respectively.

## 3. Discussion

### 3.1. Microstructural Characteristics

The reasons that the δ phase is retained are partially due to the V_2_O_5_ dopant, which acts as a phase stabilizer, as shown in Figure 7. Furthermore, further addition of V_2_O_5_ into Bi_2_O_3_ causes a mixture of phases once the solid solution limit of one single phase is exceeded. The possible stabilization mechanism is the constrained stress induced by the thermal expansion mismatch of the δ and γ phases. The stress is strong enough that the δ grains are retained unless the grains reach a critical size. Therefore, the grain sizes of less than 5 µm after 100 h annealing in these dual phase conductors are still lower than the critical size. Furthermore, the decreased number of oxygen vacancies due to the increasing V_2_O_5_ dopant may also stabilize the crystal structure.

The images of B1V and B6V samples show no obvious phase separation. Very low content of the γ phase in B1V is expected in the matrix. However, the γ grains might be very small in size or distributed randomly, but invisible by scanning electron microscope (SEM). On the other hand, B6V is dense (Figure 7) and contains only the γ phase in the solid solution of V_2_O_5_. Therefore, the image contrasts of B1V and B6V are similar, appearing homogeneous in the phases and having little residual porosity.

### 3.2. Ionic Conductivity

The conductivity mechanisms in the dual-phase conductors might be controlled by the amount of the δ phase and the oxygen vacancy concentration. The oxygen ions transported in the defective δ grains are also affected by the relative mobility of the oxygen vacancies compared to that of the electrons. When compared to pure Bi_2_O_3_, the decreased mobility of the oxygen ions results in increased activation energy, as mentioned before. The valance state of Bi ions was 3+ and 4+/5+ for V ions, respectively, resulting in less oxygen vacancies (1%) in B8V than B6V. However, the higher the concentration of the oxygen vacancies of B6V, the more possible it is that these oxygen vacancies participate in defect clustering, or arrange in order, lowering the diffusion mobility, as well as the ionic conductivity [20,21]. However, the most important factor causing B8V to have higher electrical conductivity is the continuous-linking state of δ grains formed in B8V; the δ grains are in an amount of up to 52 mol%, enough to establish a continuous network for electrical conductivity, mainly ionic and less electronic. This is likely happening at lower temperatures (<500 °C) in long-term annealed samples.

There were turning points of the electrical conductivity in B7V at 600 °C and in B8V at 550 °C. The conductivity at higher temperatures might have arisen from the small polaron mechanism [22,23]. The small polarons in these composite conductors are a combination of a negatively-charged electron and a positively-charged vacancy ($e^{-}:V_{O}^{2+}$). Lone electron pairs (LEPs) of Bi ions tend to turn toward any vacancy site nearby. The electrons are strongly trapped in this structure due to a Coulombic interaction that induces a small polarization field. As the temperature increases, the vibration and mobility of the small polarons increase. In other words, the oxygen vacancies mobilize at 550 °C or higher temperatures. The small polarons dominate the conductive mechanism of the B8V sample. As a result, the activation energy changes dramatically from 0.8 eV down to 0.2 eV at the turning points.

## 4. Materials and Experimental Procedures

### 4.1. Sample Preparation

Bi-V-O electrolytes were prepared by solid-sate reaction of powder mixtures, which were mixed by a similar process as described in our previous report [15]. The powders, Bi_2_O_3_ (99.5%, Solar Applied Materials Technology Corporation, Tainan City, Taiwan) and V_2_O_5_ (99.5%, Riedel de-Haen, Seelze, Germany) were dispersed separately in polyethylene (PE) bottles with de-ionized water and 1 wt% dispersants Ceramo D-134 (an ammonium salt of a co-polymer, Dai-ichi Kogyo Seiyaku Co., Ltd., Tokyo, Japan) based on the powder mass. The slurries of 20 vol% solid loading were ball-milled for 48 h to reach a well-dispersed condition, and then, both slurries were mixed in specified ratios. The surface potentials of the powders in diluted slurry were measured by a zeta-meter (3.0+, Zeta-Meter Inc., Staunton, VA, USA), and the particle sizes were checked by a particle sizer (MASTERSIZER 2000, Malvern Instruments, Malvern, UK).

After drying the slurries by a rotary vacuum evaporator, the dried mixtures were collected and sieved through a 1000 mesh sieve and then die pressed. The die-pressed pellets were placed in a covered Al_2_O_3_ crucible with some sacrificed powder, in which a 10 wt% V_2_O_5_-Bi_2_O_3_ mixture was placed in the corner. The interior of the crucible formed a protective vapor pressure at sintering temperatures to protect the samples from the vaporization of the Bi_2_O_3_ and V_2_O_5_ species. All of the sintered samples went through sintering at 800 °C for 1 h or the aging steps at 650, 740 or 800 °C as long as 100 h for testing mass loss. None of the following reported samples in this study experienced more than 0.5% (equivalent to 0.5 mg·h^−1^·cm^−2^) mass loss by the thermal treatments.

After sintering, some samples went through further thermal annealing at selected temperatures for 100 h to observe the mass loss. The crystalline phases nearly reached a thermodynamically-stable state, thus allowing comparison with the phases shown in the reported Bi-V-O binary phase diagrams.

### 4.2. Characterizations

Zeta-potential measurement: The Bi_2_O_3_ and V_2_O_5_ powders were dispersed in DI water with 0.5 M NaCl electrolyte. The solid concentration of the slurries was 100 ppm. The slurries were adjusted to pH values from 2 to 9 and then kept for one day to reach an equilibrium state. Before measuring the zeta-potential, the pH values of the slurries were measured again and recorded. Finally, the zeta-potentials of the powder in an aqueous condition were measured by Zeta-Meter 3.0+ (Zeta-Meter Inc.).

Thermal analysis: Differential thermal analysis (SDT Q600, TA Waters LLC, New Castle, DE, USA) was used to determine the phase transformation of the γ phase. A Pt crucible was used in DTA and TGA analysis. The operation conditions from room temperature to 1000 °C were conducted at a heating rate of 10 °C/min.

Quantitative X-ray diffraction: Crystallographic data were examined by an X-ray diffractometer (Rigaku, Tokyo, Japan) using Cu Kα radiation. Diffraction patterns were collected from the 2θ range between 20° and 60° at a scan speed of 5°·min^−1^ and in a step of 0.02°.

SEM microstructure: Back scattering electron (BSE) micrographs were used to observe the phases of the Bi-V-O compounds with a JEOL JSM6510 (JEOL, Tokyo, Japan). The amount of phases (α, γ and δ) on the SEM micrographs was quantified by an equipped image analyzing program.

Electrical and ionic conductivities: A 2-probe direct current (DC) method was used to measure the electrical conductivity (σ) of the sintered Bi-V-O samples. The pellet-shaped samples were polished, and then, Pt film was applied on both sides in an area of 0.28 cm^2^ and a thickness of ca. 100 nm. Then, Ag wire was connected on the Pt electrodes bonded by Ag paste. The sample was put into a furnace and annealed at 350 °C for 30 min for binder-burn-out and connected to a digital multimeter (7555, Yokogawa, Tokyo, Japan) testing from 400 to 650 °C at intervals of 50 °C. The output resistance can be converted into electrical conductivity (σ) by the following formula:

(2) $$\sigma =\frac{t}{RA}$$

where *t* is the thickness of the BxV sample (cm), *R* is the resistance of the sample (Ω) and *A* is the cross-section area of the sample (cm^2^).

**Figure 10:**
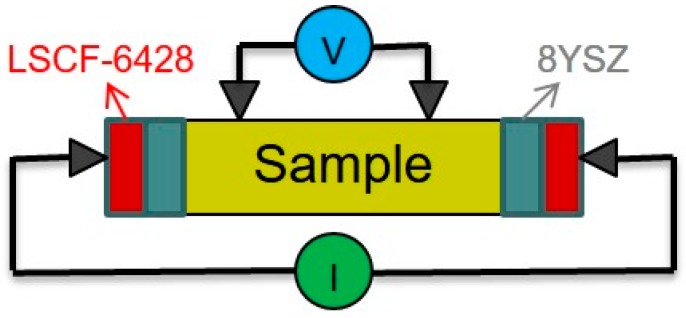

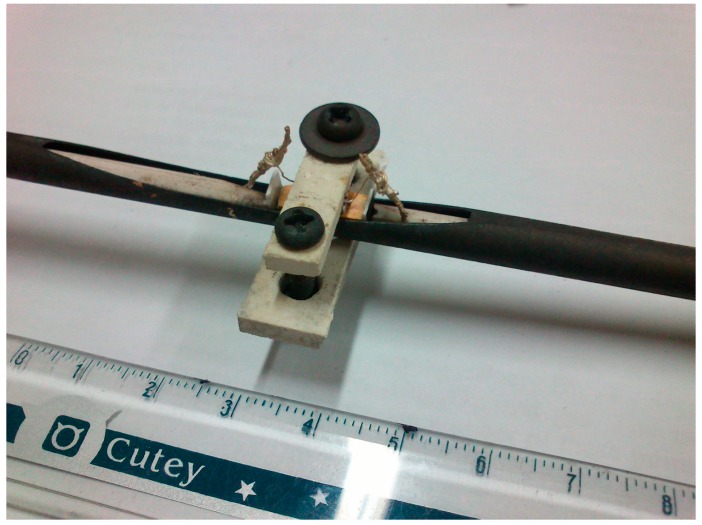
Schematic diagram and picture of the arrangement of the 4-probe DC method and tooling for ionic conductivity measurement. LSCF-6428, (La_0.6_Sr_0.4_)(Co_0.2_Fe_0.8_)O_3_; 8YSZ, 8 mol% yttria-stabilized zirconia.

A 4-probe method was used to measure the ionic conductivities of the Bi-V-O samples. The arrangement is shown in Figure 10, which is modified from that proposed by Teraoka et al. [24]. The rectangular sintered sample was placed in the middle of two dense 8YSZ (8 mol% yttria-stabilized zirconia) plates and thin (La_0.6_Sr_0.4_)(Co_0.2_Fe_0.8_)O_3_ (LSCF-6428) conductive layers. The YSZ electrodes were used to block the electrons owing to the fact that 8YSZ was totally ionic in the temperature region. Then, the electrodes were connected with Ag wires to lead out the current. Two YSZ-made tip electrodes were connected to a voltmeter (V) and closely contacted to the top surface of the sample with a fixing device, as shown in Figure 10. All contact points of the electrode to the sample would have a contact force of 1 N or more. Knowing the cross-sectional area (A), the distance between two voltage electrodes (*t*) and resistance of the sample (*R*), the ionic conductivity (σ_i_) of samples could be calculated from Equation (2). Furthermore, the transference number (*t*_i_) can be calculated as below.

*t*_i_ = σ_i_/σ (3)

## 5. Conclusions

Homogeneous Bi_1−x_V_x_O_1.5+x_ samples have been prepared by a designed colloidal process. Bi_2_O_3_ and V_2_O_5_ powders in the slurries dispersed with D-134 dispersant show iso-electric-points located at pH 3.2 and have stronger surface potentials (exceeding −30 mV) in neutral and basic conditions. The surface repulsive forces are strong enough to separate the particles in a stabilized condition for as long as a few days.

The thermal analysis results show that directly heating the powder mixture to 800 °C at a heating rate of 10 °C/min is effective for reaching a uniform microstructure. The V_2_O_5_ content is capable of forming a completely solid solution with Bi_2_O_3_ below 690 °C. The calcination step often used in other reports is not needed in the sintering of the Bi-V-O materials. The effective prevention of mass loss of Bi_2_O_3_-based samples to a level of <0.002% at 800 °C for 3 h is achieved by burying the sample in a Bi_2_O_3_-based powder bed.

The solid solution of the γ phase in the Bi-V-O system ranges from 5.7 to 6.9 mol%. When above 6.9 mol%, an additional 6:1 or 7:1 phase appears, and below 5.7 mol%, an additional α phase appears. The dual phase microstructures of B4V, B7V, B8V and B9V are induced by the V-content and long annealing at 650 °C. B6V was found by XRD to be single phase.

All B7V, B8V and B9V samples show a dual-phase microstructure, and two hold similar activation energies in the range of 0.78–0.83 eV below 550 °C, implying that the three conductors have the same conductive mechanism, i.e., oxygen-vacancy-induced conduction. At temperatures >550 °C, the activation of conduction occurs by a small polaron effect. Among the BxV samples (x ≤ 10), B8V shows the highest electrical conductivity of 0.003 S/cm at 650 °C and an ionic transference number of nearly 0.90. The transference number indicates that the BxV samples are a mixed ionic-electronic (MIE)-type conductor. 

## Figures and Tables

**Table 1:** Mass loss selected BxV compositions by different thermal annealing in air.

Sample	Sintering Temperature	Mass Sintered	Mass * after Holding at 800 °C for 100 h	Loss % in 100 h	Sublimation Rate (g/h·cm^2^)
**B4V**	800 °C/3 h	0.276 g	0.275 g	0.36%	0.0018%
**B6V**	800 °C/3 h	0.436 g	0.434 g	0.46%	0.0013%
**B8V**	800 °C/3 h	0.417 g	0.415 g	0.48%	0.0017%
**B15V**	800 °C/3 h	0.428 g	0.427 g	0.23%	0.0016%

* The mass loss of the sample was in an accuracy range ±0.0001 g of the balance.

**Figure A1:**
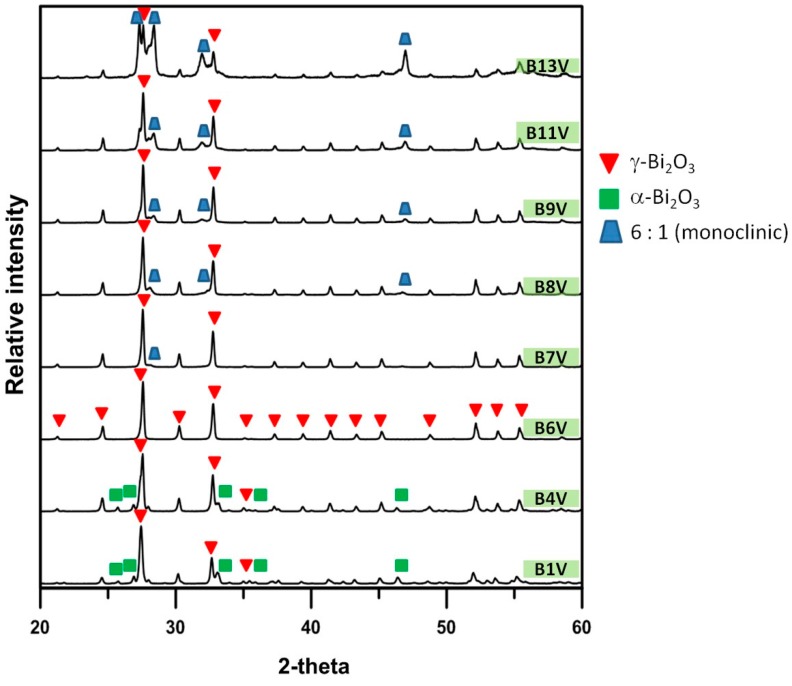
XRD patterns of B1V–B13V die-pressed mixtures, annealed at 600 °C for 100 h.

**Figure A3:**
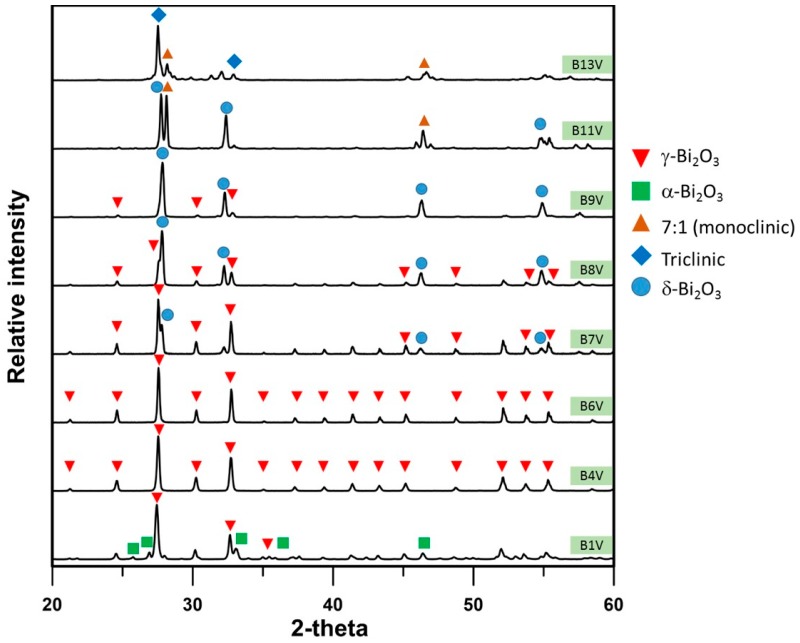
XRD patterns of B1V–B13V die-pressed mixtures, annealed at 800 °C for 100 h.
